# Preoperative Adrenal Artery Embolization in a Pediatric Patient with Bilateral Pheochromocytomas: A Case Report

**DOI:** 10.7759/cureus.106334

**Published:** 2026-04-02

**Authors:** Claudia Moya Ochoa, Eduardo Bravo Rius, Gloria González, Daniela Mistretta Solorza, Leonardo Álvarez Aros

**Affiliations:** 1 Interventional Radiology, Hospital de Niños Dr. Luis Calvo Mackenna, Santiago, CHL; 2 Pediatric, General, and Thoracic Surgery, Cleveland Clinic Children's Hospital, Cleveland, USA; 3 Anesthesiology, Hospital de Niños Dr. Luis Calvo Mackenna, Santiago, CHL; 4 Radiology, Hospital Clínico Universidad de Chile, Santiago, CHL

**Keywords:** adrenal embolization, case report, catecholamine crisis, paediatric interventional radiology, paraganglioma, pediatric surgery, pediatric surgery, pheochromocytoma

## Abstract

Pheochromocytomas in pediatric patients pose significant perioperative risks due to catecholamine-induced hemodynamic instability, and preoperative arterial embolization has been proposed as an adjunctive strategy to reduce surgical risk; however, experience in bilateral pediatric cases remains limited. We describe the case of a 12-year-old male with incidentally detected bilateral pheochromocytomas measuring 5.0 cm (right) and 3.0 cm (left) and markedly elevated 24-hour urinary metanephrines (24,150 μg/24 hours). After adequate pharmacologic preparation with alpha- and beta-adrenergic blockade, the patient underwent bilateral preoperative transarterial adrenal artery embolization using calibrated microspheres, detachable microcoils, and a liquid embolic agent, achieving near-complete devascularization of both glands. Despite pre-procedure preparation, hypertensive crises occurred during angiography; however, the staged laparoscopic bilateral adrenalectomy was completed with fewer and less severe intraoperative hemodynamic events than anticipated.

The postoperative course was uneventful, with discharge on postoperative day seven and initiation of lifelong glucocorticoid and mineralocorticoid replacement therapy. Preoperative transarterial adrenal embolization may serve as a valuable adjunct in the multidisciplinary management of pediatric patients with large or bilateral pheochromocytomas to reduce intraoperative hemodynamic instability, and this technique warrants further investigation to define optimal patient selection, technical approaches, and long-term oncologic and endocrine outcomes.

## Introduction

Pheochromocytomas and paragangliomas (PPGLs) are rare catecholamine-secreting neuroendocrine tumors arising from chromaffin cells of the adrenal medulla or extra-adrenal sympathetic ganglia, with an estimated incidence of approximately 0.3 cases per million persons per year [1]. Although PPGLs primarily affect adults, 10% to 20% of cases are diagnosed in childhood, typically around a mean age of 11 years [1]. Excessive catecholamine release can precipitate life-threatening complications, including severe hypertensive crises, malignant arrhythmias, catecholamine-induced cardiomyopathy, and cardiogenic shock [2]. Complete surgical resection remains the definitive treatment; however, perioperative management is particularly challenging due to the risk of profound hemodynamic instability, which is further compounded in pediatric patients by physiologic variability and difficulties in medication titration [3,4].

Adequate preoperative alpha-adrenergic blockade is the cornerstone of perioperative preparation, with beta-blockers added only after effective alpha-blockade has been established to avoid paradoxical hypertension. Nevertheless, achieving optimal hemodynamic control with pharmacologic therapy alone may be insufficient in patients with large or highly vascular tumors [4]. Preoperative transarterial embolization has been proposed as an adjunct strategy to reduce tumor vascularity, minimize intraoperative blood loss, facilitate surgical dissection, and attenuate catecholamine release [5]. However, its role in pediatric PPGLs remains poorly defined, with published experience largely limited to isolated case reports [3,5,6]. Here, we report a pediatric case of bilateral pheochromocytomas managed with preoperative adrenal artery embolization as part of a coordinated multidisciplinary strategy and discuss the potential role of this approach in optimizing perioperative outcomes in this challenging population.

## Case presentation

Patient information and medical history

A 12-year-old male with no significant past medical history was admitted following an uneventful laparoscopic appendectomy performed at an outside hospital. During postoperative monitoring, the patient was found to have persistent hypertension (blood pressure 157/97 mmHg), prompting an evaluation for secondary causes. There was no family history of pheochromocytoma, paraganglioma, or known hereditary tumor syndromes.

Clinical findings

On physical examination, the patient appeared well-developed and well-nourished. Vital signs indicated a heart rate of 120 beats per minute, blood pressure of 162/94 mmHg, respiratory rate of 35 breaths per minute, a temperature of 35.8°C, and body weight of 38 kg. Cardiovascular assessment revealed a regular rhythm with normal heart sounds and no murmurs. The abdomen was soft, non-tender, and without palpable masses. No cutaneous stigmata of neurofibromatosis or other phakomatoses were observed. The remainder of the physical examination was unremarkable.

Diagnostic assessment

**Figure 1:**
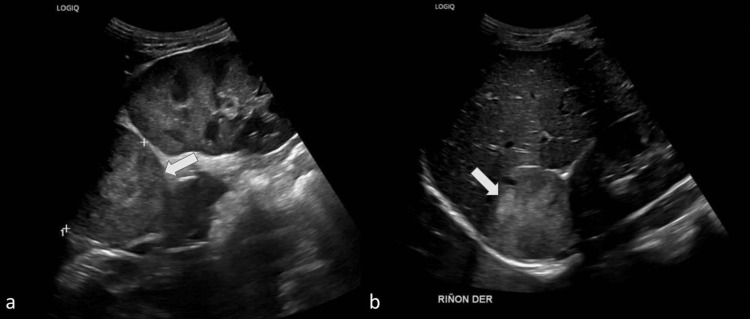
Abdominal ultrasound as part of a secondary hypertension workup demonstrated an incidental right adrenal mass (white arrows)

**Figure 2:**
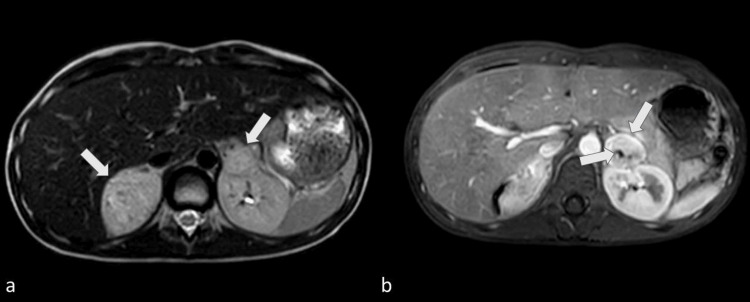
Abdominal MRI demonstrated bilateral adrenal masses, measuring 5.4 × 4.0 × 5.0 cm (right) and 3.0 × 2.5 × 3.3 cm (left), with no evidence of local invasion or lymphadenopathy. a: T2-weighted turbo spin-echo images show bilateral hyperintense adrenal lesions (white arrows); b: Gadolinium-enhanced T1-weighted images reveal early heterogeneous enhancement and areas of cystic degeneration (white arrows), suggestive of bilateral pheochromocytomas

Initial abdominal ultrasonography identified a mass at the upper pole of the right kidney (Figure 1). Subsequent contrast-enhanced abdominal MRI revealed bilateral adrenal masses with imaging characteristics consistent with pheochromocytomas, measuring 5.0 cm on the right and 3.0 cm on the left in their greatest dimension. Both lesions demonstrated heterogeneous T2-weighted signal hyperintensity and avid gadolinium enhancement (Figure 2).

**Figure 3:**
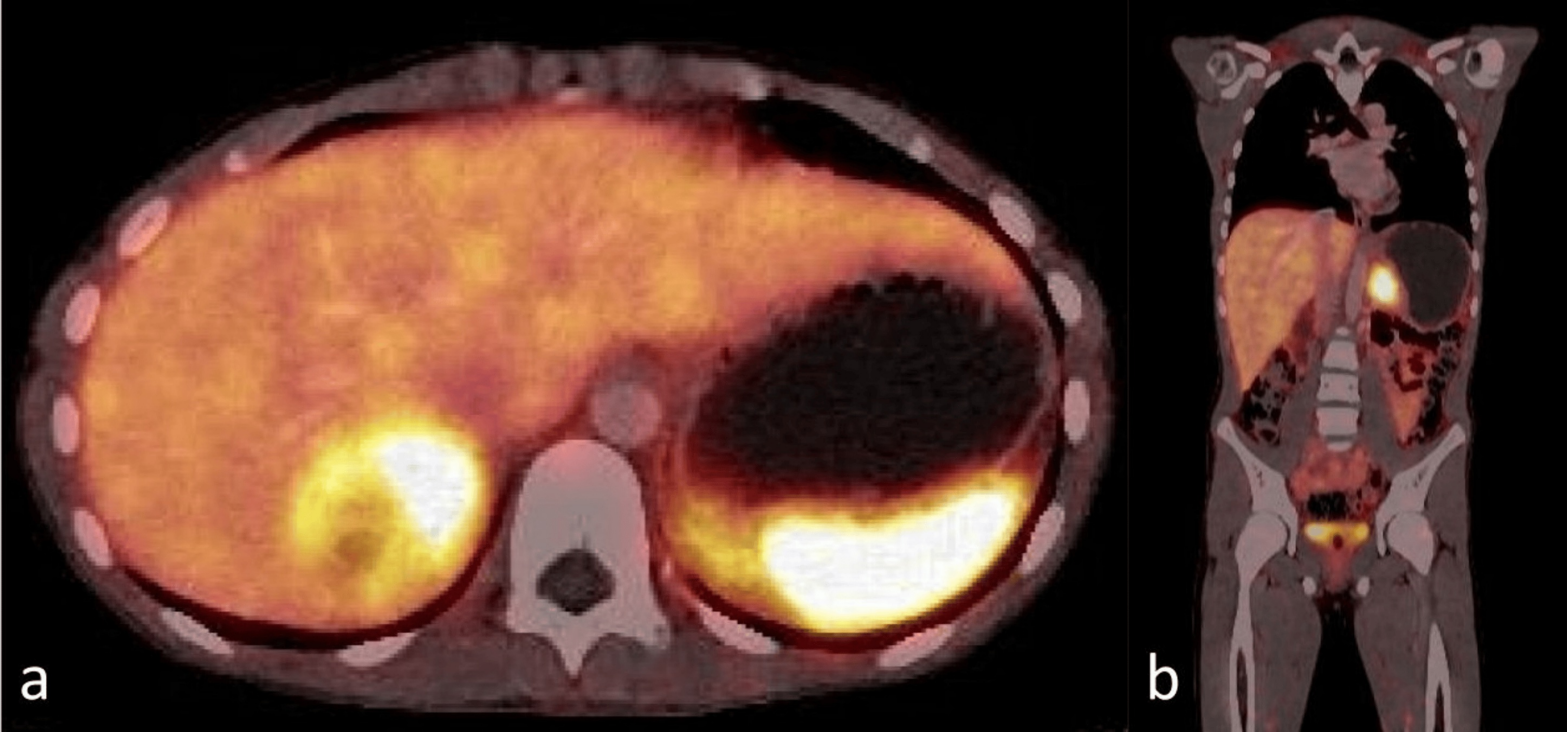
Whole-body ¹⁸F-aluminium fluoride (AlF)-NOTA-octreotide PET-CT revealed bilateral heterogeneous adrenal masses with markedly elevated radiotracer uptake, reflecting somatostatin receptor overexpression a: Right adrenal mass (5.5 cm; standardized uptake value (SUV)max 15.9); b: Left adrenal mass (3.2 cm; SUVmax 15.2), consistent with bilateral pheochromocytoma. There was no evidence of secondary dissemination.

Biochemical evaluation revealed markedly elevated 24-hour urinary metanephrines: normetanephrine 24,150 μg/24 hours (normal reference range 32-343 μg/24 hours) (Table 1). Whole-body 18F-fluorodeoxyglucose PET-CT demonstrated no evidence of metastatic disease (Figure 3). Genetic evaluation testing for hereditary PPGL syndromes was requested. However, due to financial constraints, genetic testing could not be completed and remains pending.

**Table 1:** Preoperative laboratory results

Parameter		Value	Reference values
Hematology	Hematocrit	27.7%	35.0-45.0
	Hemoglobin	8.9 g/dL	12.0-16.0
	White blood cells	14,900 /μL	4,000-14,000
	Neutrophils	70.6%	33.0-59.0
	Lymphocytes	21.7%	33.0-52.0
	Platelets	423,000 /μL	150,000-450,000
Coagulation	INR	1.1	
	Activated partial thromboplastin time (aPTT)	28.8 s	22.0-42.0
Biochemistry	Creatinine	0.54 mg/dL	0.20-0.70
	Magnesium	1.6 mg/dL	1.6-2.2
	Glucose	87 mg/dL	74-126
	Total bilirubin	0.42 mg/dL	< 1.0
	Alkaline phosphatase	106 U/L	106
	Alanine transaminase (ALT)/aspartate aminotransferase (AST)	65/48 U/L	10-54/15-40
	Calcium	9.1 mg/dL	9.0-11.0
	Albumin	3.1 g/dL *	4.0-6.0
Inflammatory markers	C-reactive protein (CRP)	116 mg/L *	<10
	Procalcitonin	1.61 ng/mL	
	Lactate	11.5 mg/dL	< 20
Endocrine/metabolic	Urine normetanephrine	24,150 μg/24h	32-343 ug/24 hours

Therapeutic interventions

Pharmacologic Preparation

The patient initially presented with persistent hypertension, with blood pressure values up to 162/94 mmHg, and sinus tachycardia reaching 120 beats per minute. Alpha-adrenergic blockade was initiated with doxazosin on day three, followed on day four by the introduction of continuous intravenous labetalol infusion to achieve blood pressure and heart rate control, resulting in normotension and normocardia.

During treatment, the patient developed an elevation in liver transaminases (aspartate aminotransferase (AST)/alanine transaminase (ALT) up to 105/103 U/L, respectively), raising suspicion of an adverse drug reaction to labetalol. Consequently, beta-blockade was discontinued for three days, resulting in a decrease in liver enzyme levels. However, this was accompanied by a recurrence of tachycardia, reaching up to 120 beats per minute at rest, along with elevated blood pressure. Given the suspected drug-related hepatotoxicity, beta-blockade was subsequently transitioned to propranolol, which achieved adequate heart rate control with good tolerance.

Preoperative Transarterial Adrenal Embolization

**Figure 4:**
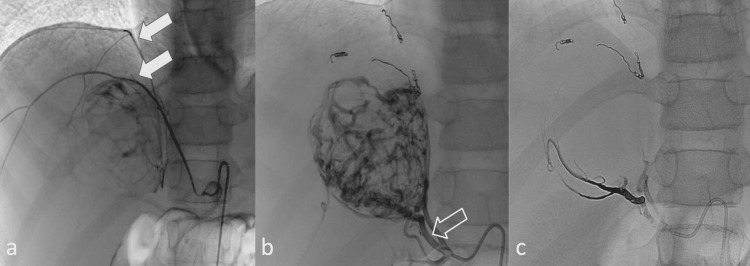
Preoperative embolization of the right adrenal tumor a: Inferior phrenic artery angiography demonstrating vascular supply to the cephalic portion of the tumor via two principal branches (white arrows). Both branches were embolized distally with microcoils, and the proximal segment with 400-µm EmboSphere (Merit Medical Systems Inc., South Jordan, UT, USA) and microcoils. b: The right middle adrenal artery (arrow) was embolized with EmboSphere and Onyx 18 (Medtronic, Minneapolis, MN, USA) c: Post-embolization angiographic control

**Figure 5:**
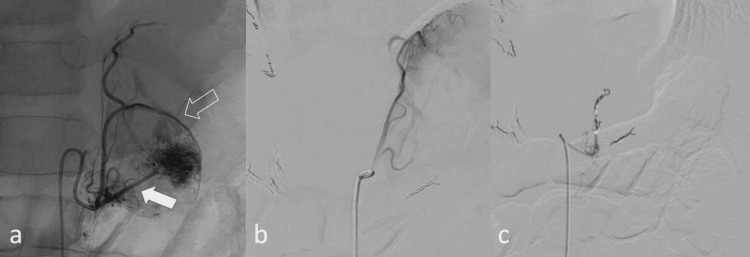
Preoperative embolization of the left adrenal tumor a: Selective angiography demonstrating vascular supply to the left adrenal tumor via the ipsilateral middle adrenal artery (white arrow) and an accessory branch originating from the inferior phrenic artery (arrow outline) b: Digital subtraction angiography (DSA) of the inferior phrenic artery after embolization of the middle adrenal artery using EmboSphere (Merit Medical Systems Inc.) and microcoils c: Completion angiography of the inferior phrenic artery following embolization with microcoils, demonstrating near-complete devascularization of the tumor’s principal arterial supply.

The embolization procedure was conducted under multimodal general anesthesia utilizing sevoflurane, remifentanil, and dexmedetomidine, with continuous invasive arterial blood pressure monitoring via a radial arterial catheter. Central venous access was established to facilitate the administration of vasoactive medications. Via a right common femoral artery approach using a 4 French sheath, selective abdominal aortography followed by renal and adrenal artery angiographies was performed. Selective catheterization demonstrated arterial supply to both adrenal tumors arising predominantly from the middle adrenal arteries and branches of the inferior phrenic arteries (Figure 4, panels a and b; Figure 5, panels a and b).

During selective angiography and contrast administration, multiple episodes of severe hypertensive crisis were observed, with peak systolic blood pressure reaching 160/80 mm/Hg, indicative of acute catecholamine release triggered by vascular manipulation. These events were managed with continuous intravenous infusions of labetalol and sodium nitroprusside, along with the deepening of anesthesia. Embolization of tumor-feeding arterial branches was performed using a combination of 400 μm EmboSphere (Merit Medical Systems Inc., South Jordan, UT, USA), 2 mm × 8 cm microcoils, and Onyx 18 liquid embolic agent (Medtronic, Minneapolis, MN, USA). The procedure was continued until near-complete devascularization was achieved, as confirmed by final angiographic assessment, with only minimal residual collateral flow observed (Figure 4, panel c; Figure 5, panel c). Following the completion of embolization, a transient hypotensive phase occurred, attributed to an acute reduction in circulating catecholamines. Low-dose norepinephrine infusion was initiated and rapidly tapered over three hours as hemodynamic stability was restored.

Surgical resection

Approximately 24 hours following embolization, staged bilateral laparoscopic adrenalectomy was performed. The left adrenal gland was resected first, followed by the right.

Left Adrenalectomy

The patient was placed in right lateral decubitus with a Trendelenburg tilt. Access was achieved via Veress needle (13 mmHg pneumoperitoneum) and a 5 mm umbilical port. Three additional 5 mm trocars were placed in the epigastrium, left flank, and paramedian line. Adhesions between the omentum, colon, and the left abdominal wall were lysed. The left renal fossa was identified, the peritoneum was incised, and the colon was reflected. Short gastric vessels were divided to access the splenic angle. The renal capsule was dissected to expose the renal hilum, identifying the renal artery, superior polar artery, and renal vein. The adrenal vein was identified and electrocoagulated. Dissection proceeded superiorly along the tumor. The previously embolized middle adrenal artery with its coils was identified and divided. Intraoperatively, the tumor appeared edematous and exhibited less vascularity than typically observed in non-embolized pheochromocytomas (Figure 5). Hypertensive episodes occurred during manipulation of the left adrenal tumor; however, these events were fewer in number and less severe than anticipated based on the preoperative catecholamine elevation. Bleeding from the tumor was controlled with two 5 mm clips without complications. The specimen was placed in an endoscopic bag and left in the abdominal cavity for later retrieval. Ports were closed with Vicryl 4/0 and Monocryl 5/0 (Ethicon Inc., Raritan, NJ, USA).

Right Adrenalectomy

The patient was repositioned in left lateral decubitus. The same umbilical port was used with an additional 5 mm trocar. Two further 5 mm trocars were placed in the right paramedian flank and right iliac fossa. The liver was elevated via the epigastric port. The right renal fossa was dissected, freeing the kidney between the duodenum and the renal fossa. The renal capsule was dissected to expose the right kidney, adrenal gland, and a firm mass measuring 7×8.5 cm. The cava, middle hepatic vein, and suprahepatic vein were identified. Dissection proceeded superiorly and parallel to the tumor mass. The previously embolized middle and superior adrenal arteries were identified and divided. On the right side, where embolization had been successful, no hypertensive crises occurred during manipulation. Dissection continued until complete resection was achieved. The specimen was introduced into an Endo Catch™ (Medtronic) bag. A Pfannenstiel incision was made for peritoneal access, and both specimens, including the previously abandoned left tumor, were extracted and sent for pathological analysis. Hemostasis was confirmed satisfactory. The peritoneum and ports were closed in layers.

Histopathology

**Figure 6:**
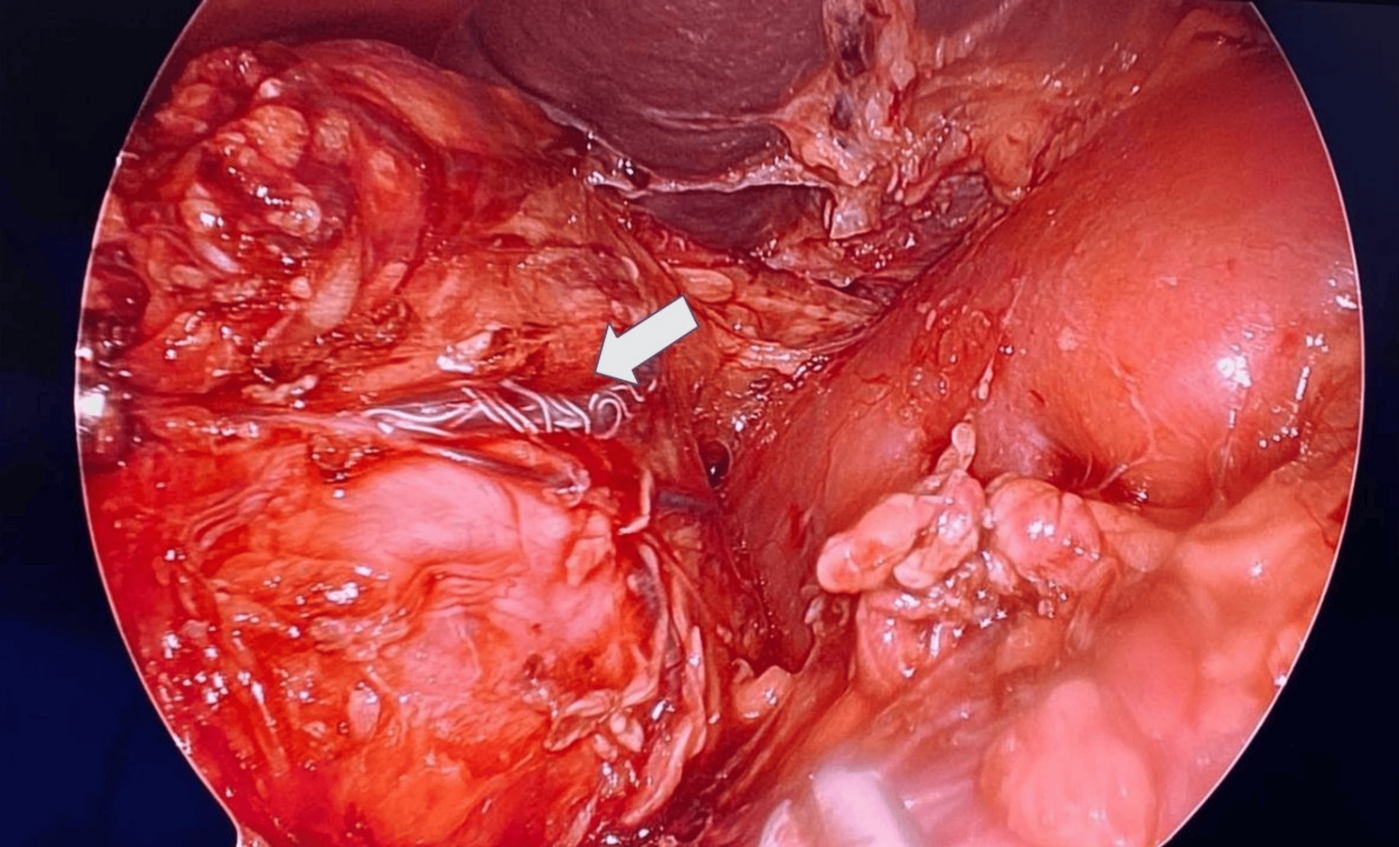
Intraoperative findings Laparoscopic view of a right pheochromocytoma, showing the adrenal tumor with a coil placed in one of its main feeding arteries (white arrow).

**Figure 7:**
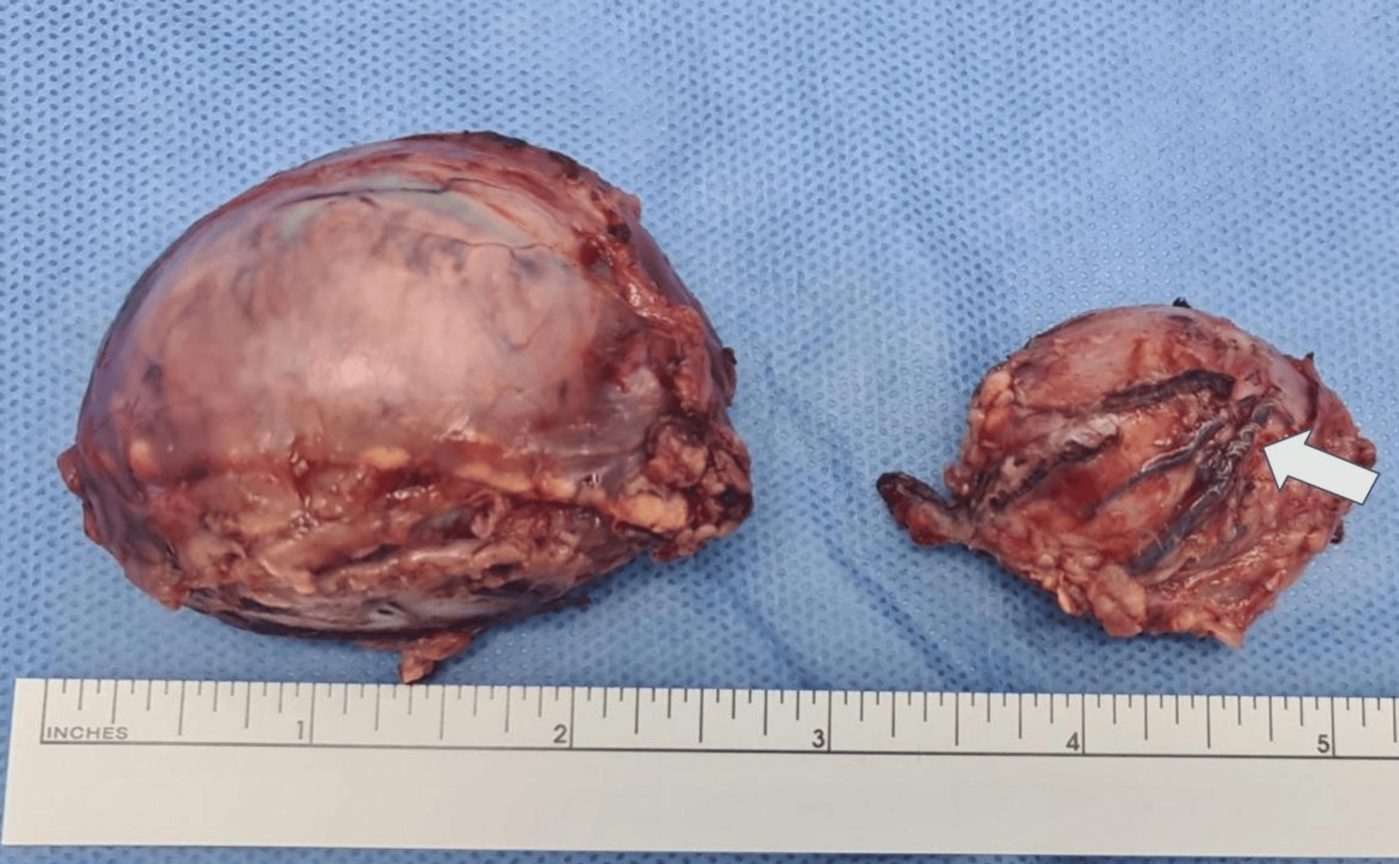
Surgical specimen of bilateral pheochromocytomas Excised adrenal tumors with characteristic morphology and vascular features post-resection, with coils visible in the left tumor (white arrow).

The procedure was completed without incidents. Both specimens were sent for deferred biopsy. Gross pathological examination revealed right and left adrenal masses measuring 5.6 × 5.0 × 3.8 cm and 4.0 × 3.6 × 2.0 cm, respectively, both exhibiting characteristic golden-yellow cut surfaces (Figures 6-7). Histopathological analysis confirmed bilateral pheochromocytomas. Surgical margins were negative, with no evidence of capsular breach or extra-adrenal extension. The histological features of both lesions demonstrated characteristics associated with malignant potential according to the Pheochromocytoma of the Adrenal Gland Scaled Score (PASS), with a PASS score of 6 for the smaller left adrenal tumor and 10 for the larger right adrenal tumor.

Follow-up and outcomes

The postoperative course was uneventful. Following bilateral adrenalectomy on postoperative day (POD) one, the patient initially experienced transient hypotension but subsequently demonstrated progressive hemodynamic stabilization. Blood pressure normalized to 98/48 mmHg, corresponding to the 25th-50th percentile, and he did not require vasopressor support beyond the immediate postoperative period. Postoperative biochemical evaluation demonstrated normalization of catecholamine secretion, with plasma and urinary metanephrines within the reference ranges. The patient was started on lifelong glucocorticoid and mineralocorticoid replacement therapy: hydrocortisone was gradually tapered from 50 mg/m²/day to 13 mg/m²/day, and fludrocortisone was initiated at a dose of 0.1 mg/day, in accordance with the pre-established endocrine management plan. Extensive education regarding adrenal crisis prevention was provided.

The patient was discharged in stable condition on POD seven. At the 12-month follow-up, he remains normotensive without antihypertensive medications, with no biochemical evidence of recurrent disease. The patient has been referred for genetic counseling and long-term surveillance according to current guidelines. See Appendix A for their perspective on the treatment plan and outcome. Table 2 features the timeline of diagnostic tests, treatments, and follow-up initiated for the patient.

**Table 2:** Timeline of the patient's treatment

Date	Event
Day zero	Initial presentation with postoperative persistent hypertension following laparoscopic appendectomy
Day one	Abdominal ultrasound revealing right adrenal mass, and abdominal MRI confirming bilateral adrenal masses compatible with pheochromocytomas
Day two	Biochemical testing: markedly elevated 24-hour urinary metanephrines
Day three	Initiation of alpha-adrenergic blockade with doxazosin
Day five	Addition of beta-adrenergic blockade with propranolol
Day 12	PET-CT scan showed no evidence of metastatic disease
Day 19	Bilateral preoperative adrenal embolization
Day 20	Staged bilateral laparoscopic adrenalectomy
Day 27	Hospital discharge
12 months	Follow-up visits with a stable clinical course

## Discussion

This case illustrates the potential utility of preoperative transarterial adrenal embolization as an adjunctive strategy in the multidisciplinary management of a pediatric patient with bilateral pheochromocytomas. While alpha-adrenergic blockade remains the foundation of preoperative preparation, it can be particularly challenging to achieve optimal hemodynamic control in children with large, hypervascular, or bilateral tumors [4].

Several factors supported the decision to pursue preoperative embolization in this case. First, the bilateral nature of the tumors necessitated complete bilateral adrenalectomy, which significantly increased the procedural complexity and duration of catecholamine exposure. Second, the markedly elevated urinary metanephrines (24,150 μg/24 hours) indicated high secretory activity and thereby elevated perioperative risk. Third, angiographic assessment confirmed hypervascular tumors with substantial arterial supply from multiple feeding vessels. This approach aligns with the broader principle that adequate preoperative stabilization, rather than urgent or emergent resection, is associated with significantly lower perioperative morbidity and mortality in pheochromocytoma [7]. In patients where pharmacological blockade alone proves insufficient to achieve hemodynamic stability, adjunctive strategies such as preoperative embolization may serve as a bridge to a more controlled surgical intervention.

The observed clinical course suggests potential benefits from the embolization strategy. Hypertensive crises during the embolization procedure itself are a recognized and expected complication, reflecting the same physiological mechanism responsible for intraoperative hemodynamic instability during surgical resection: massive catecholamine release triggered by tumor manipulation [8]. Despite this, intraoperative hemodynamic events during surgical resection were fewer and less severe than reported in comparable cases managed without preoperative embolization [9,10]. Furthermore, the reduced tumor vascularity facilitated surgical dissection, resulting in minimal blood loss.

These observations are consistent with prior reports describing preoperative embolization in pheochromocytoma. Hrabovsky et al. [9] first reported this technique in a pediatric patient over four decades ago, demonstrating significant reductions in urinary catecholamine levels following embolization. Subsequent case reports and small series have similarly reported improvements in hemodynamic stability and surgical outcomes [10,11,12]. Notably, to our knowledge, this represents the first reported case of bilateral preoperative adrenal artery embolization prior to bilateral adrenalectomy for pheochromocytoma in a pediatric patient (Table 3). In all previously reported cases, embolization was performed unilaterally, even when bilateral disease was present. The bilateral approach employed in this case was driven by the symmetric hypervascularization of both tumors and the markedly elevated catecholamine burden, and it may have contributed to the favorable intraoperative hemodynamic profile observed during resection of the right adrenal gland, where no hypertensive crises were recorded. This observation suggests that bilateral embolization may offer incremental benefit over unilateral embolization in cases of bilateral hypervascular pheochromocytoma, though further experience is needed to validate this hypothesis.

**Table 3:** Summary of reported cases of preoperative arterial embolization in pheochromocytoma/paraganglioma NR: Not reported; TAE: Transcatheter arterial embolization; CgA: Chromogranin A; VMA: Vanillylmandelic acid; PVA: Polyvinyl alcohol; ACC: Adrenocortical carcinoma; vHL: von Hippel-Lindau disease; PASS: Pheochromocytoma of the Adrenal Gland Scaled Score

Characteristic	Hrabovsky et al., 1982 [9]	Di Daniele et al., 2012 [10]	Sormaz et al., 2017 (case 1) [11]	Sormaz et al., 2017 (case 2) [11]	Sormaz et al., 2017 (case 3) [11]	El Mostapha et al., 2021 [12]	Present case
Age (in years)/sex	13/male	30/female	22/female	24/female	42/female	16/male	12/male
Diagnosis	Pheochromocytoma	Extra-adrenal paraganglioma (pelvic)	Pheochromocytoma + paraganglioma (von Hippel-Lindau (vHL) disease)	Adrenocortical carcinoma	Perivascular epithelioid cell neoplasms (PEComa)	Pheochromocytoma	Bilateral pheochromocytoma
Tumor location	Left suprarenal	Left adnex (extra-adrenal)	Right adrenal + left paraganglioma	Right adrenal	Right adrenal	Left adrenal	Bilateral adrenal
Tumor size	Not reported (NR)	7 cm	8 cm (right)/1.8 cm (left)	18 cm	17 cm	60×72×75 mm	5 cm (right) / 3 cm (left)
Embolization (laterality)	Unilateral	Unilateral	Unilateral (right)	Unilateral	Unilateral	Unilateral	Bilateral
Embolic agent	Gelfoam	NR	Polyvinyl alcohol (PVA) + polyzene-F microspheres	Polyzene-F microspheres	Polyzene-F microspheres	NR	Embospheres, microcoils, and Onyx 18
Interval transcatheter arterial embolization (TAE) → surgery	Five days	Same admission	24 hours	24 hours	24 hours	NR	24 hours
Embolization-related complications	None	None	Hypertensive attack (managed with sodium (Na) nitroprusside)	None	None	None	Hypertensive attack (managed with Na nitroprusside and labetalol)
Hemodynamic improvement post-TAE	Yes (↓ BP, ↓ urinary catecholamines)	Yes (↓ chromogranin A (CgA), ↓ urinary vanillylmandelic acid (VMA), and catecholamines)	Yes (↓ BP)	N/A (non-functional)	N/A (non-functional)	Yes (↓ BP)	Yes (↓ BP)
Intraoperative findings	No complications; left nephrectomy required	No complications	↓ Collateral vessels; no major bleeding	↓ Parasitic venous caliber; no major bleeding	↓ Hypervascularity; no major bleeding	Stable hemodynamics	↓ Hypervascularity; no major bleeding
Surgical outcome	Successful; normotensive at 1 year	Successful; normotensive at follow-up	Successful; discharged day seven	Successful; discharged day four	Successful; discharged day five	Successful; normotensive post-op	Successful; normotensive at follow-up
Pathology confirmed	Pheochromocytoma	Paraganglioma	Pheochromocytoma (right) + paraganglioma (left)	Adrenocortical carcinoma (ACC) (partial capsular invasion, 16 cm)	PEComa (18 cm)	Pheochromocytoma (PASS 2)	Bilateral pheochromocytoma (PASS 6 left / PASS 10 right)

The physiological rationale is sound: reducing tumor blood supply may directly decrease catecholamine synthesis and release, while tumor devascularization may attenuate the catecholamine surge typically observed during surgical manipulation. However, several considerations temper enthusiasm for this approach. First, the embolization procedure itself carries inherent risks, including non-target embolization, post-embolization syndrome, and paradoxically severe catecholamine crises during catheter manipulation, as observed in our patient. Second, the evidence supporting this strategy remains limited to case reports and small retrospective series, precluding definitive conclusions about its efficacy. Third, patient selection criteria, optimal timing relative to surgery, and preferred embolic agents have yet to be standardized.

Strengths and limitations

This case report has notable strengths. It highlights a rare clinical scenario: bilateral pheochromocytoma in a pediatric patient managed through a multimodal, multidisciplinary approach. Detailed descriptions of the embolization procedure are provided, offering valuable insights for future clinical practice. Nevertheless, important limitations must be acknowledged. As a single case report, it cannot establish causality or definitive conclusions regarding the efficacy of preoperative embolization. The subjective characterization of 'fewer and less severe' hemodynamic events lacks objective quantification, which limits the strength of the observed association. Additionally, information regarding genetic testing and long-term follow-up remains incomplete. The applicability and generalizability of our experience to other patients and care settings are inherently limited. 

## Conclusions

Preoperative transarterial adrenal embolization may serve as a valuable adjunct in the multidisciplinary management of carefully selected pediatric patients with large, hypervascular, or bilateral pheochromocytomas. In this case, embolization was associated with improved perioperative hemodynamic control and facilitated surgical resection. However, the procedure carries inherent risks and should be performed only by experienced interventional teams in close collaboration with surgical, anesthesia, and endocrinology specialists.

These findings support further investigation of preoperative embolization in pediatric PPGLs, with particular attention to defining optimal patient selection criteria and procedural techniques. Future studies should prioritize the quantification of hemodynamic improvements following embolization, the assessment of endocrine function after bilateral adrenalectomy, and the implementation of protocols for adrenal crisis prevention in the perioperative period. Long-term follow-up data are also needed to evaluate hormonal recovery and adrenal insufficiency outcomes in pediatric patients undergoing bilateral resection. Given the rarity of this condition, prospective registries or multicenter collaborations will likely be necessary to generate sufficient evidence to establish standardized management guidelines.

## Appendices

Appendix A

Patient and Caregiver Perspective

The parents of the 12-year-old boy described the bilateral pheochromocytoma diagnosis as terrifying, fearing major surgery and risks to their son's life. The suggestion of preoperative bilateral adrenal embolization offered cautious hope as a less invasive option to control his severe hypertensive crises and lower surgical risk.

They reported the procedure was well tolerated, with minimal discomfort and rapid recovery. Their son returned to normal activities within days, without further crises or complications. They expressed deep gratitude for the team's clear communication and careful planning, which eased their anxiety. They view it as a significant advancement to have such procedural interventions available in the public health care system.
